# Increase in Self-Injury as a Method of Self-Harm in Ghent, Belgium: 1987-2013

**DOI:** 10.1371/journal.pone.0156711

**Published:** 2016-06-01

**Authors:** Nikita Vancayseele, Gwendolyn Portzky, Kees van Heeringen

**Affiliations:** Department of psychiatry and medical psychology, Ghent University, Ghent, Belgium; University of Oxford, UNITED KINGDOM

## Abstract

**Background:**

Self-harm is a major health care problem and changes in its prevalence and characteristics can have important implications for suicide prevention. The objective was to describe trends in the epidemiology of self-harm based on emergency department (A&E departments) visits over a 26-year period in Ghent, Belgium.

**Methods:**

We analyzed data on all self-harm presentations from the three large general hospitals in Ghent between 1987 and 2013. We investigated trends in prevalence (events by year per 100.000), methods and alcohol use.

**Results:**

Rates of self-harm steadily decreased during the 26-year study period. In general female rates of self-harm were higher than male rates. The mean patient age was 35 years. The most commonly used method of self-harm was self-poisoning by means of an overdose of medication (80.8%), followed by cutting (10.2%) and hanging (4.2%). Psychotropics (including antidepressants, benzodiazepines, barbiturates and other tranquilizers) were the most frequently used drugs (74.5%). A proportional increase in the use of self-injurious methods in self-harm was highly significant, more specifically in the use of hanging, jumping from heights and the use of other violent methods such as the use of firearms, jumping before a moving object or other traffic related injury.

**Conclusion:**

This epidemiological study showed an increase in the use of high-lethality methods in self-harm which has important implications for suicide prevention. As restrictions in the availability of these methods are difficult or impossible to achieve, prevention programmes will have to emphasize the role of thorough psychosocial assessment and adequate follow-up care of self-harm patients.

## Introduction

It is estimated that more than 800 000 people worldwide die by suicide annually [1]. There thus is a great need for suicide prevention programmes, which have been implemented in many countries in the world [2]. As suicide can be the consequence of multiple causal and contributory factors, such prevention programmes are commonly multifaceted, targeting both the general population and specific risk groups [3].

Individuals with a history of self-harm constitute a risk group of particular importance for suicide prevention. Self-harm occurs 10 to 20 times more frequently than suicide, and a history of self-harm is a major risk factor for suicide, being present in at least 40% of suicides [4–7] with in many cases an episode of self-harm shortly before their fatal act [8, 9]. Careful monitoring of self-harm thus is warranted to help identify aspects of self-harming behaviours that are relevant to suicide prevention and service provision. The long-term monitoring of self-harm may thus be helpful in evaluating national suicide prevention programmes [10]. Moreover, long-term monitoring may provide indications of the extent to which societal changes such as the economic recession may have an impact on the occurrence of self-harming behaviours. For example, the registry conducted by the National Registry of Deliberate Self harm in Ireland shows in 2013 a decrease in the national rate of self-harm for the third subsequent year, when the recession diminished [11].

Studies of long-term trends in rates of self-harm are scarce. Available data come from a limited number of countries (Belgium, Canada, Germany, Italy, Norway, Sweden, the UK and the US). These long-term monitoring studies show changes in rates, in the characteristics of people presenting to general hospitals following self-harm and the nature of their self-harm acts [12–21]. The majority of episodes of self-harm presented to hospitals involve self-poisoning with medicines, yet the used medicines show changes over time reflecting changes in prescribing practices and the availability of drugs [22, 23]. In general, these long-term monitoring studies of self-harm show declining rates, with some studies providing evidence of proportionally increasing rates of self-injury versus self-poisoning [15, 19, 24]. As the choice of method may be associated with suicidal intent and risk of subsequent suicide such a change in methods of self-harm is of particular importance for the prevention of suicide. The use of more lethal methods of self-harm such as firearms and hanging is associated with higher suicidal intent than the use of self-cutting and self-poisoning [25]. Strong significant associations are found for several methods of self-injury, relative to self-poisoning, and suicide by self-injury. Risks of suicide are between 2 and 6-fold for cutting (parts of the body other than wrist/arms), hanging or asphyxiation, use of CO/other gas and traffic-related self-injury [9, 26–28]. Previous studies have documented a positive association between alcohol use and (repetition of) self-harm [6, 29, 30]. Almost half of those presenting to the A&E department after an episode of self-harm have consumed alcohol in the period prior to the act [29, 29, 31]. Therefore, we need to continuously monitor trends in self-harm including the characteristics of both patients and their self-harm acts.

A monitoring system for self-harm has been in place in the three general hospitals in Ghent, which started in 1987, and thus provides a unique dataset for the long-term epidemiological surveillance of self-harm. The current study aimed, first, at describing trends in rates of self-harm over a 26-year period, i.e. between 1987 and 2013, in Ghent (Belgium). Secondly, trends in methods for self-poisoning and self-injury, including the involvement of alcohol, were studied.

## Methods

### Setting, study design and participant selection

The study was undertaken in the A&E departments of the three general hospitals in Ghent. Data on demographic and method-related characteristics of self-harm patients and acts were collected for all individuals (age 15 or older) who presented with an episode of self-harm to the A&E department of the University hospital (1987–2013) of Ghent and two general hospitals in Ghent (since 1996 till 2013) for the 26-year period from 1 January 1987 to 31 December 2013.

Between 1987 and 1994 self-harm was defined according to Hawton and Catalan (1987) [32] and included deliberate self-poisoning and self-injury. The term ‘deliberate self-poisoning’ was used to describe the deliberate ingestion of more than the prescribed amount of medical substances, or the ingestion of substances never intended for human consumption, irrespective of whether harm was intended. ‘Deliberate self-injury’ was used to describe any intentional self-inflicted injury, irrespective of the apparent purpose of the act. Since 1994, self-harm was defined as “an act with nonfatal outcome, in which an individual initiates a deliberate, well-considered, and unusual behaviour, that without intervention of another will lead to self-harm or destruction, or when an individual deliberately takes a substance in a higher quantity then subscribed or generally suitable doses, with the intention by means of actual or expected physical consequences to initiate desired changes” [33].

Demographic and clinical data on each episode were collected by clinicians and nurses using standardized forms. Through scrutiny of the records of the A&E department and the psychiatric department of the University hospital of Ghent, information was also available on patients who were admitted to the hospital but who were not seen by a psychiatrist of for whom no data sheet was collected due to early discharge, refusal of assessment, or unavailability of staff.

### Demographics, clinical characteristics and rates

Data for this study included gender, age, date of self-harm, method and use of alcohol. The data on methods were coded according to ICD-10 codes. Self-injury involved violent methods (X70-X77; X78-X84), while self-poisoning involved less violent methods (X60 to X69). Self-harm episodes involving alcohol alone were not included unless accompanied by other types of self-poisoning or self-injury. Rates were calculated as the number of people (aged 15+) per 100 000 inhabitants in Ghent for each year.

### Data-analysis

Statistical analyses were performed using SPSS22. The primary analysis was a descriptive summary for self-harm. The chi square test for trend (linear by linear association) was used to test the level of significance of changes over the period 1987–2013. Groups were compared using chi-square tests. P < 0.05 was considered significant.

### Ethics statement

Ethical approval for data collection on self-harm was obtained from the Ethical Committee of the University Hospital Ghent according to the principles expressed in the declaration of Helsinki. The Ethics Committee gave approval to collect patient data without obtaining informed patient consent, this because the data were analyzed anonymously.

## Results

### Demographics

During the 26-year study period, i.e. from 1 January 1987 to 31 December 2013, the monitoring system reported 8 692 episodes of self-harm aged 15+ years in the three A&E departments in Ghent. Age and gender were known for 8 377 persons (3.7%, n = 315 were missing) (Table 1). The majority of self-harm patients were female (56.0%). The mean age for both genders was 35.5 years. More than the half of the patients (n = 4 676, 55.8%) were 35 years or younger. The largest number of females was in the 20–24 age group (n = 733, 15.6%), and the largest number of males was in the 25–29 age group (n = 591, 16.0%).

**Table 1:** Distribution of self-harm in Ghent by patient characteristics: 1987–2013.

	N	%		N	%
**Episodes** (a)	8 684				
**Males**	3 821	44.0%			
**Females**	4 863	56.0%			
**Males by age group**	3 685 (b)		**Females by age group**	4 692 (c)	
**15–19**	297	8.1%		509	10.8%
**20–24**	561	15.2%		733	15.6%
**25–29**	591	16.0%		650	13.9%
**30–34**	512	13.9%		581	12.4%
**35–39**	471	12.8%		534	11.4%
**40–44**	439	11.9%		532	11.3%
**45–49**	308	8.4%		419	8.9%
**50–54**	181	4.9%		258	5.5%
**55–59**	111	3.0%		168	3.6%
**60–64**	71	1.9%		89	1.9%
**65–69**	42	1.1%		79	1.7%
**70+**	101	2.7%		140	3.0%

^(a)^ Plus 8 episodes where gender was not known, thus total number of episodes 8 692

^(b)^ Plus 136 males whose age was not known (total number of males 3 821)

^(c)^ Plus 171 females whose age was not known (total number of females 4 863)

### Rates of self-harm (1987–2013)

**Fig 1:**
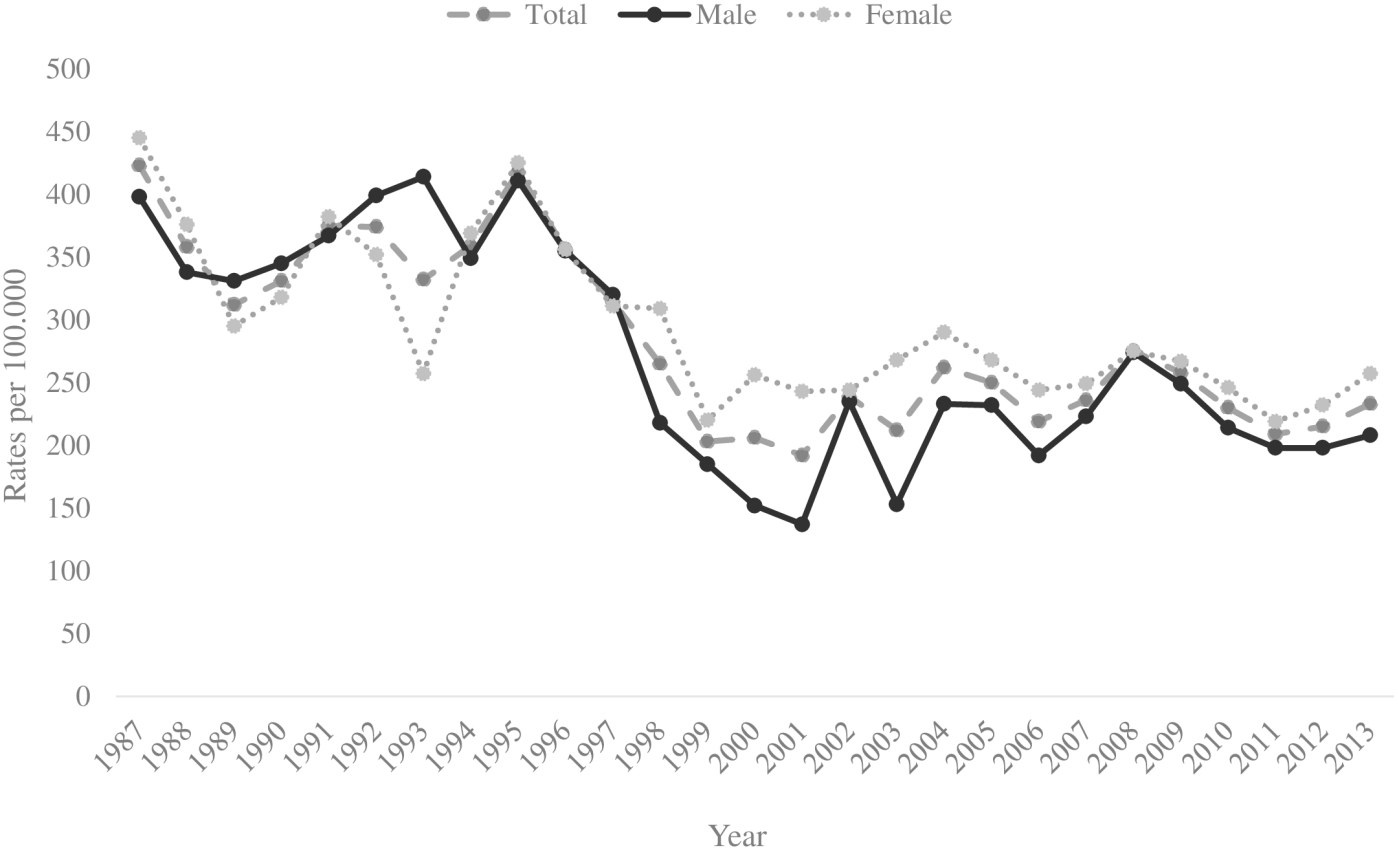
Self-harm (person based) rates in Ghent 1987–2013.

Trends in rates of self-harm are shown in Fig 1. The rates per 100 000 (95% CI) fluctuated over the 26-year period from 1987 to 2013, but the total rate declined between 1987 and 2013 significantly (χ^2^(1) = 55.21, *p* < 0.001) from 423 (95% CI 380–470) to 233 (95% CI 200–260) per 100 000 inhabitants. The decline in rates over the 26-year period was greater in males (47.7%) than females (42.2%).

Decreases in person based self-harm rates for Ghent were seen in both genders during the first three years of the study period. In the period from 1987 to 1989 the rate of males decreased significantly (χ^2^(1) = 6.18, *p* < 0.05) respectively from 398 (95% CI 360–440) to 331 (95% CI 300–370) and the rate of females decreased significantly (χ^2^(1) = 30.52, *p* < 0.001) from 445 (95% CI 410–490) to 295 (95% CI 260–330). Rates in both genders then rose significantly until 1995. Rates among males increased with 19.1% from 345 (95% CI 310–380) in 1990 to 411 (95% CI 370–450) in 1995 (χ^2^(1) = 5.78, *p* < 0.05), while rates among females increased with 33.6% from 318 (95% CI 280–350) in 1990 to 425 (95% CI 390–470) in 1995 (χ^2^(1) = 15.47, *p* < 0.001). These increases were followed by decreasing rates till 2001, especially in males (significant decrease by 61.4% (χ^2^(1) = 96.83, *p* < 0.001) from 355 (95% CI 320–390) in 1996 to 137 (95% CI 120–160) in 2001. Among females, rates declined with 31.7% (χ^2^(1) = 21.38, *p* < 0.001) from 356 (95% CI 320–390) in 1996 to 243 (95% CI 210–280) in 2001. Between 2002 and 2008 the rates in both genders steadily, but not significantly increased again, i.e. among males with 16.6% from 235 (95% CI 210–270) to 274 (95% CI 240–310) and among females with 12.7% from 244 (95% CI 220–280) to 275 (95% CI 240–310). During the last five years of the study period the rates decreased slightly and non-significantly from 249 (95% CI 220–280) in 2009 to 208 (95% CI 180–240) in 2013 among males (i.e. a decrease of 16.5%) and from 267 (95% CI 240–300) in 2009 till 257 (95% CI 230–290) in 2013 among females (i.e. a decrease of 3.7%).

Overall, female rates of self-harm were significantly higher than male rates. In 1989, 1990, 1992 and 1997 the male rate was not significantly higher than the female rate, while in 1993 the male rate was significantly higher than the female rate (i.e. 414 and 257 (χ^2^(1) = 36.86, *p* < 0.001).

### Trends in methods of self-harm

Among the 8 692 episodes of self-harm, 6 804 episodes (78.3%; 95% CI 77.4%-79.1%) involved self-poisoning only, while 1 279 episodes (14.7%; 95% CI 13.9%-15.4%) involved self-injury only and 459 episodes (5.3%; 95% CI 4.8%-5.8%) involved both self-poisoning and self-injury. For 150 episodes the used method (1.7%; 95% CI 1.5%-2.0%) was unknown.

**Fig 2:**
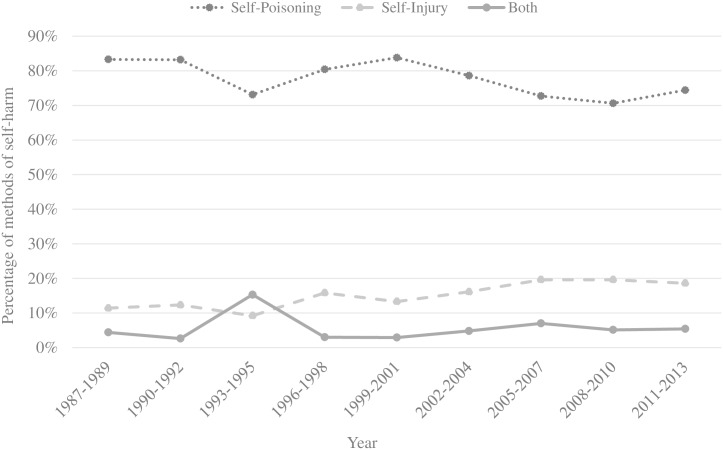
Trends in method of self-harm, Ghent 1987–2013.

Fig 2 shows the trends in the proportions of episodes using self-poisoning and self-injury while self-harming during the study period. From 1987 to 2013 the vast majority of events involved self-poisoning. The use of self-poisoning only (overdose medication and/or self-poisoning with pesticides or gases and unspecified chemicals and noxious substances) as a method of self-harm decreased during the study period with 9.3% (χ^2^ for trend: 49.83, *p* < 0.001). More specifically, there was a strong decrease in the use of an overdose medication from 83.0% (95% CI 80.6%-85.2%) in 1987–1989 to 77.4% (95% CI 74.3%-80.2%) in the period 2011–2013 (χ^2^ for trend: 26.32 *p* < 0.001) and a decrease in the use of pesticides or gases and unspecified chemicals and noxious substances from 5.5% (95% CI 4.3%-7.0%) in 1987–1989 to 2.8% (95% CI 1.9%-4.2%) in the period 2011–2013 (χ^2^ for trend: 38.25, *p* < 0.001). During the 26 years of monitoring, females (85.7%; 95% CI 84.7%-86.6%) used significantly more commonly self-poisoning with an overdose of medication as method than males (74.6%; 95% CI 73.2%-75.9%; χ^2^ (1) = 169.98, *p* < 0.001). Males (4.1%; 95% CI 3.5%-4.7%) used significantly more commonly self-poisoning with pesticides or gases and unspecified chemicals and noxious substances as method than females (3.1%; 95% CI 2.6%—3.6%; χ^2^ (1) = 6.24, *p* < 0.05).

During the study period, psychotropic drugs (including benzodiazepines, barbiturates, antidepressants, other tranquillizers) and unspecified drugs (including antibiotics and anti-hypertensive drugs) were involved in self-poisoning in 74.5% (n = 5 237) and 14.8% (n = 1 042), respectively. Non-narcotic analgesics (mainly involving paracetamol) were used in 979 episodes (13.9%), more commonly in females (15.5%; 95% CI 14.5%-16.7%) than in males (11.6%; 95% CI 10.5%-12.8%; χ^2^ (1) = 21.55, *p* < 0.001). A combination of drugs from different classes was used in 17.7% of episodes (n = 1 244).

The use of methods involving self-injury increased during the study period (χ^2^ for trend: 60.33, *p* < 0.001) from 11.4% (95% CI 9.6%-13.4%) in 1987–1989 to 18.6% (95% CI 16.0%-21.5%) in the period 2011–2013. More specifically, there was a strong increase in the use of hanging from 2.6% (95% CI 1.8%-3.8%) in 1987–1989 to 5.1% (95% CI 3.8%-6.9%) in the period 2011–2013 (χ^2^ for trend: 14.94, *p* < 0.001), jumping from heights from 1.1% (95% CI 0.6%-1.9%) in 1987–1989 to 3.3% (95% CI 2.3%-4.9%) in the period 2011–2013 (χ^2^ for trend: 53.60, *p* < 0.001) and the use of other violent methods from 1.0% (95% CI 0.6%-1.8%) in 1987–1989 to 3.2% (95% CI 2.2%-4.7%) in the period 2011–2013 (χ^2^ for trend: 38.10, *p* < 0.001), including the use of firearms, jumping before a moving object or other traffic-related injury. Hanging was significantly more common between 50 and 59 years (6.7%; 95% CI 5.1%-8.7%) than among other age groups (3.9%; 95% CI 3.5%-4.4%; χ^2^ (1) = 12.29, *p* < 0.001) and significantly more frequent among males (5.8%; 95% CI 5.1%-6.6%) than among females (2.7%; 95% CI 2.3%-3.2%; χ^2^ (1) = 53.19, *p* < 0.001). Drowning was significantly more common among patients aged 60 or more (4.4%; 95% CI 3.0%-6.5%) than in other age groups (1.3%; 95% CI 1.1%-1.6%; χ^2^ (1) = 14.56, *p* < 0.001). Young people between 15 and 19 years (4.2%; 95% CI 3.0%-5.8%) used significantly more frequent jumping from heights as a method than people in other age groups (2.4%; 95% CI 2.1%-2.7%; χ^2^ (1) = 9.90, *p* < 0.01). Self-injury using a sharp object (i.e. cutting, 10.2%; 95% CI 9.5%-10.8%) was the most commonly used violent method, without statistically significant changes over time. Cutting was significantly more common between 20 and 29 years (12.2%; 95% CI 11.0%-13.6%) than in other age groups (9.6%; 95% CI 8.9%-10.4%; χ^2^ (1) = 13.20, *p* < 0.001) and decreased with increasing age (χ^2^ for trend: 23.51, *p* < 0.001). Significant more males (13.1%; 95% CI 12.1%-14.2%) than females (7.9%; 95% CI 7.1%-8.7%) used cutting as a method (χ^2^ (1) = 64.15, *p* < 0.001). During the 26 years of monitoring, males (19.8%; 95% CI 18.6%-21.1%) used significantly more commonly self-injury as a method than females (10.7%; 95% CI 9.9%-11.6%; χ^2^ (1) = 140.39, *p* < 0.001). Table 2 shows trends in the method used of self-harm during the study period.

**Table 2:** Method of self-harm (1987–2013).

	Year		Males		Females
Method of self-harm		N	% (95% CI)	N	% (95% CI)
**Self-poisoning**					
**Overdose medication**	1987–1989*	365	76.0 (72.0, 79.6)	526	88.7 (85.9, 91.0)
	1990–1992*	411	74.6 (70.8, 78.1)	504	88.0 (85.0, 90.3)
	1993–1995*	406	79.1 (75.4, 82.4)	443	86.4 (83.1, 89.1)
	1996–1998*	496	76.0 (72.5, 79.1)	696	86.2 (83.7, 88.5)
	1999–2001**	225	80.9 (75.9, 85.1)	399	87.9 (84.6, 90.6)
	2002–2004*	261	76.1 (71.3, 80.3)	471	85.5 (82.3, 88.2)
	2005–2007*	301	70.7 (66.2, 74.8)	473	82.0 (78.6, 84.9)
	2008–2010*	181	65.3 (59.6, 70.7)	252	80.3 (75.5, 84.3)
	2011–2013*	202	67.6 (62.1, 72.6)	401	83.5 (80.0, 86.6)
**Poisoning**	1987–1989	27	5.6 (3.9, 8.1)	32	5.4 (3.9, 7.5)
	1990–1992	38	6.9 (5.1, 9.3)	27	4.7 (3.3, 6.8)
	1993–1995	34	6.6 (4.8, 9.1)	33	6.4 (4.6, 8.9)
	1996–1998	10	1.5 (0.8, 2.8)	16	2.0 (1.2, 3.2)
	1999–2001	2	0.7 (0.2, 2.6)	3	0.7 (0.2, 1.9)
	2002–2004	8	2.3 (1.2, 4.5)	8	1.5 (0.7, 2.8)
	2005–2007	14	3.3 (2.0, 5.4)	14	2.4 (1.5, 4.0)
	2008–2010	8	2.9 (1.5, 5.6)	8	2.5 (1.3, 5.0)
	2011–2013	14	4.7 (2.8, 7.7)	8	1.7 (0.9, 3.3)
**Self-injury**					
**Hanging**	1987–1989*	23	4.8 (3.2, 7.1)	5	0.8 (0.3, 1.2)
	1990–1992*	19	3.4 (2.2, 5.3)	3	0.5 (0.2, 1.5)
	1993–1995	43	8.4 (6.3, 11.1)	37	7.2 (5.3, 9.8)
	1996–1998**	27	4.1 (2.9, 6.0)	12	1.5 (0.9, 2.6)
	1999–2001	10	3.6 (2.0, 6.5)	9	2.0 (1.1, 3.7)
	2002–2004*	23	6.7 (4.5, 9.9)	11	2.0 (1.1, 3.5)
	2005–2007**	33	7.7 (5.6, 10.7)	23	4.0 (2.7, 5.9)
	2008–2010**	24	8.7 (5.9, 12.6)	13	4.1 (2.4, 7.0)
	2011–2013	21	7.0 (4.6, 10.5)	19	4.0 (2.6, 6.1
**Drowning**	1987–1989	8	1.7 (0.9, 3.3)	10	1.7 (0.9, 3.1)
	1990–1992	5	0.9 (0.4, 2.1)	8	1.4 (0.7, 2.7)
	1993–1995	4	0.8 (0.3, 2.0)	6	1.2 (0.5, 2.5)
	1996–1998	9	1.4 (0.7, 2.6)	10	1.2 (0.7, 2.3)
	1999–2001	4	1.4 (0.6, 3.6)	6	1.3 (0.6, 2.9)
	2002–2004	2	0.6 (0.2, 2.1)	7	1.3 (0.6, 2.6)
	2005–2007	8	1.9 (1.0, 3.7)	10	1.7 (0.9, 3.2)
	2008–2010	5	1.8 (0.8, 4.2)	9	2.9 (1.5, 5.4)
	2011–2013	8	2.7 (1.4, 5.2)	12	2.5 (1.4, 4.3)
**Cutting**	1987–1989*	62	12.9 (10.2, 16.2)	36	6.1 (4.4, 8.3)
	1990–1992*	67	12.2 (9.7, 15.2)	34	5.9 (4.3, 8.2)
	1993–1995**	81	15.8 (12.9, 19.2)	52	10.1 (7.8, 13.1)
	1996–1998*	91	13.9 (11.5, 16.8)	65	8.1 (6.4, 10.1)
	1999–2001**	34	12.2 (8.9, 16.6)	27	5.9 (4.1, 8.5)
	2002–2004	39	11.4 (8.4, 15.2)	53	9.6 (7.4, 12.4)
	2005–2007	52	12.2 (9.4, 15.7)	57	9.9 (7.7, 12.6)
	2008–2010	29	10.5 (7.4, 14.6)	22	7.0 (4.7, 10.4)
	2011–2013	45	15.1 (11.4, 19.5)	36	7.5 (5.5, 10.2)
**Alcohol**	1987–1989*	174	36.3 (32.1, 40.1)	144	24.3 (21.0, 27.9)
	1990–1992**	145	26.3 (22.8, 30.2)	105	18.3 (15.4, 21.7)
	1993–1995**	133	25.9 (22.3, 29.9)	98	19.1 (15.9, 22.7)
	1996–1998**	208	31.9 (28.4, 35.5)	193	23.9 (21.1, 27.0)
	1999–2001**	109	39.2 (33.7, 45.1)	124	27.3 (23.4, 31.6)
	2002–2004**	121	35.3 (30.4, 40.5)	150	27.2 (23.7, 31.1)
	2005–2007**	150	35.2 (30.8, 39.9)	147	25.5 (22.1, 29.2)
	2008–2010*	95	34.3 (29.0, 40.1)	63	20.1 (16.0, 24.8)
	2011–2013*	111	37.1 (31.8, 42.7)	115	24.0 (20.4, 28.0)

* = p < .001

** = p < .05

Between 1993 and 1995 the use of self-poisoning decreased, while the combined use of self-poisoning and self-injury increased, particularly involving the combination of cutting or hanging and medication.

### Involvement of alcohol

The use of alcohol before or during self-harm was more common among males (32.6%; 95% CI 31.2%-34.1%) than among females (23.4%; 95% CI 22.3%-24.6%; (χ^2^ (1) = 91.16, *p* < 0.001). During the study period, the use of alcohol before or during self-harm increased significantly among males (χ^2^ for trend: 7.53, *p* < 0.01). There was no significant change over the years in the use of alcohol among females. Over the 26 years, the use of alcohol was most common in the 35–54 years age groups.

## Discussion

Using a unique long-term dataset of self-harm presentations to the three general hospitals in Ghent, this study investigated trends in the rates and characteristics of self-harm between 1987 and 2013. Overall rates of self-harm appeared to peak in 1995 and then declined in both genders. There were obvious changes in the methods used for self-harm during the study period with a significant proportional increase in the use of self-injury as the method of self-harm. More specifically, there was a strong increase in the use of hanging, jumping from heights and the use of other violent methods including the use of firearms, jumping before a moving object or other traffic-related injury. Cutting was most common between 20–29 years and people aged 60 and older used commonly drowning as method. Overall, the use of self-injurious methods increased from 11 to 19%, or approximately from one in ten episodes to one in five episodes of self-harm.

Preceding a discussion of implications of the present findings for suicide prevention potential methodological limitations need to be addressed. We were able to use data on a large sample of self-harm patients, which were collected by means of a consistent monitoring system during a prolonged period of time in the three major hospitals in the area. The reliability of the findings is supported by the comparability of demographic characteristics to those from other epidemiological studies of self-harm [15, 16, 18–21, 29]. However, the data most probably refer to a selected group of self-harm patients. The sample did not include individuals who self-harmed but did not present to hospital. There are patients who self-harmed but did not need medical attention or are treated by a general practitioner without referral to an A&E department. This indicates a possible under reporting of the real number of people who self-harm in the study area.

The marked decline in rates of self-harm since 1995 coincides with a change in the definition of self-harm in the hospitals since 1994 as described in the method section. However, as the decline in rates of self-poisoning was much more outspoken than the change in rates of self-injury it is unlikely that the decrease in rates is attributable to the change of definition. It is possible that local prevention activities have contributed to the decrease in self-harm rates. For example, in 1995, we demonstrated a near-significant effect of home visits by a community nurse in case of non-compliance with referral to outpatient aftercare among self-harm patients after discharge from the A&E departments involved in the current study, on repetition of self-harm [34]. Since 1997, this activity has been integrated in a suicide prevention programme in which community mental health services target high-risk groups such as self-harm patients. The prevention programme includes the availability of help for self-harm patients within 48 hours after a self-harm episode, educational campaigns targeting health care professionals (e.g. general practitioners, nurses and police officers) by organizing seminars and workshops about suicide prevention and assistance in developing suicide prevention strategies in schools and companies. The Flemish government has implemented two subsequent suicide-prevention programmes during the study period. The first programme ran from 2006 to 2010 [35]. The second Flemish suicide prevention programme was launched in 2012, aiming at a 20.0% decrease in suicide rates in 2020 when compared to 2000 [36]. Suicide rates in Flanders have stabilized between 2009 and 2012, thus not showing the substantial increases, which became apparent in surrounding countries such as The Netherlands during this period, and which are thought to be related to the economic turmoil since 2008 [37]. The extent to which the suicide prevention programme has countered the negative impact of the economic problems and thus contributed to the stabilisation of suicide rates and the decrease in self-harm rates in times of economic problems remains to be elucidated. The continued monitoring of self-harm will demonstrate whether the second prevention programme is associated with a further decrease in rates of self-harm. Other targets of the Flemish suicide prevention programme such as improved media reporting of suicide may also have contributed to the decline. There may have been other reasons for the declining rates of self-harm, such as changes in help-seeking behaviour including the increased use of counseling services (chat and forums), telephone support [38] and internet websites for help and support [39].

The increased proportional use of self-injury in self-harm may be due to changes in patterns of hospital presentations of self-harm over time. For example, the proportional increase in self-injury may be due to a decrease in hospital presentations of self-poisoning. Such a change in hospital presentations cannot be ruled out, but might have been reflected by a stronger decrease in rates in females than in males, as females more commonly self-harm by means of self-poisoning than males. As this was not the case, it appears that there indeed is a relative increase in self-injurious methods of self-harm during the study period. In addition, a similar trend in the use of self-injury has been found in other studies [15, 31]. Given the up to 5-fold increased risk of subsequent suicide following self-injury when compared to a non-violent method such as self-poisoning [40, 41] and the increased suicidal intent associated with the use of highly lethal methods of self-injury [25], this finding may be of particular relevance for suicide prevention and e.g. the A&E department staff when making decisions about follow-up care [25].

The current findings have important implications for suicide prevention. First, with regard to methods used in self-harm the data show that the medication used in self-poisoning mainly consists of psychotropic drugs. This is in contrast with findings in other countries such as the UK in which shows paracetamol and paracetamol-containing compounds are the most commonly used drugs in self-poisoning [29]. Such differences most probably reflect an effect of availability of drugs whether or not via prescriptions. This issue therefore needs to be addressed in educational campaigns and training programmes targeting physicians. Such training programmes may lead to marked effects in reducing suicide rates [42, 43]. A reduction in the accessibility of medication may indeed contribute to suicide prevention [44]. In Ireland, the withdrawal of a prescription-only analgesic compound was associated with a marked reduction in the rate of intentional drug overdose presentations to hospital involving the drug [45]. Restricting the availability of medication has led to marked reductions in suicidal behaviour in England, Wales, Scotland and Scandinavia [46–48]. Secondly, the relative increase in self-injury, due to increases in the use of hanging, jumping from heights and the use of other violent methods such as the use of firearms, jumping before a moving object or other traffic related injury, poses an important challenge to prevention. These methods are generally available and a restriction in the access to these methods appears to be difficult or even impossible to achieve [49].

The findings may also have implications for the A&E departments in the general hospitals [31]. Psychosocial assessment following self-harm, is a necessary starting point for preventive interventions. One of the strategies in the Flemish suicide prevention programme [36], aims at increasing the use of a standardized instrument to assess self-harm patients in general hospitals. As this may lead to reduced rates of subsequent repetition of self-harm [50, 51]. In case when it is impossible to conduct a full psychosocial assessment, the A&E department staff should be aware that the use of highly lethal methods of self-injury such as the use of firearms and hanging is associated with high suicidal intent and a high risk of suicide so that intensive follow-up care and risk reduction measures are indicated for these individuals [9, 25, 26].

In conclusion, using data from A&E departments in major general hospitals this long-term monitoring study of the occurrence and characteristics of self-harm shows decreasing rates of self-harm between 1987 and 2013, however with a significant increase in the use of self-injurious methods. Given the fact that self-poisoning continues to be the most common method of self-harm, prevention strategies targeting physicians and their prescription practices continue to be important components of suicide prevention strategies. The proportional strong increase in the use of self-injurious methods in self-harm poses however new challenges to public health approaches in suicide prevention. In any case, the current findings underline the necessity of adequate management of self-harm patients in A&E departments of general hospitals as a very important component of suicide prevention programmes.
